# Pharmacological management of cherubism: A systematic review

**DOI:** 10.3389/fendo.2023.1104025

**Published:** 2023-03-14

**Authors:** Pierre-Emmanuel Cailleaux, André Luís Porporatti, Martine Cohen-Solal, Natacha Kadlub, Amélie E. Coudert

**Affiliations:** ^1^ Université Paris Cité, Institut National de la Santé et de la recherche médicale (Inserm) U1132 Biologie de l'os et du cartilage (BIOSCAR), Paris, France; ^2^ Faculté or Unité de formation et de recherche (UFR) d’Odontologie, Laboratoire de Neurobiologie Oro-Faciale (EA 7543), Université Paris Cité, Paris, France; ^3^ Faculté or Unité de formation et de recherche (UFR) de Médecine, Université Paris Cité, Institut National de la Santé et de la recherche médicale (Inserm) U1132 Biologie de l'os et du cartilage (BIOSCAR), Hôpital Lariboisière, Paris, France; ^4^ Faculté or Unité de formation et de recherche (UFR) de Médecine, Université Paris Cité, Inserm 1138, Centre de Recherche des Cordeliers, Paris, France; ^5^ Faculté or Unité de formation et de recherche (UFR) d’Odontologie, Université Paris Cité, Institut National de la Santé et de la recherche médicale (Inserm) U1132 Biologie de l'os et du cartilage (BIOSCAR), Paris, France

**Keywords:** cherubism, pharmacological treatment, drug, systematic review, management – healthcare

## Abstract

**Objective:**

The aim of this systematic review was to determine if there exists an efficacious drug treatment for cherubism, based on published studies.

**Methods:**

This systematic review included observational case studies reporting pharmacological management of cherubism. We developed specific search strategies for PubMed (including Medline), ScienceDirect, Web of Science. We evaluated the methodological quality of the included studies using criteria from the Joanna Briggs Institute’s critical appraisal tools.

**Results:**

Among the 621 studies initially identified by our search script, 14 were selected for inclusion, of which five were classified as having a low risk of bias, four as having an unclear risk, and five a high risk. Overall, 18 cherubism patients were treated. The sample size in each case study ranged from one to three subjects. This review identified three types of drugs used for cherubism management: calcitonin, immunomodulators and anti-resorptive agents. However, the high heterogeneity in case reports and the lack of standardized outcomes precluded a definitive conclusion regarding the efficacy of any treatment for cherubism.

**Conclusions:**

The present systematic review could not identify an effective treatment for cherubism due to the heterogeneity and limitations of the included studies. However, in response to these shortcomings, we devised a checklist of items that we recommend authors consider in order to standardize the reporting of cherubism cases and specifically when a treatment is given toward identification of an efficacious cherubism therapy.

**Systematic review registration:**

https://www.crd.york.ac.uk/prospero/display_record.php?ID=CRD42022351044, identifier CRD42022351044.

## Introduction

Cherubism (OMIM #118400) is a rare autosomal dominant bone disease characterized by progressive and painless bilateral and symmetrical osteolysis of the jaw bones. To date, about 500 cases (1) have been reported in the international literature, in both sexes and in various ethnic groups. Cherubism is considered to be an autoinflammatory disease which affects craniofacial bones only (2). The natural course follows a three-step evolution in children: expansion, stabilization and regression. The first signs occur in early childhood and then progress, slowing down after 7 years of age, stabilizing during puberty, and regressing thereafter (3). Cherubism is caused by gain-of-function mutations of the *SH3BP2* gene, which codes for the adaptor protein SH3BP2. Various forms of cherubism have been described, from asymptomatic to aggressive forms with orbital damage, or even lethal cases (4, 5). Severity grades were described according to different classifications (6–9) based on the X-ray images.

In the early stages of cherubism, especially in the aggressive form, cervical lymphadenopathy has been described (10, 11). Histologically, lesions are composed of a multinucleated giant-cell granuloma of dense non-neoplastic fibrous connective stroma with fibroblasts and multinucleated giant osteoclast-like cells, such as in reparative granuloma or brown tumor of hyperparathyroidism (12–14). Exploration is limited to imaging and histology. Standard blood count and bone biomarkers (such as serum calcium and phosphate concentrations, TSH, FSH, LH, PTH, PTHrP, T4 and T3 hormone, calcitonin, osteocalcin) remain within the normal range in numerous case reports (15). As expected in this disease with important bone resorption, some authors have noted changes in bone turnover biomarkers. An increase in bone-specific alkaline phosphatase level and a slight increase in urinary deoxypyridinoline have also been described (16).

Although the causative mutation of cherubism has been known since 2001 (17), both the pathogenetic mechanism responsible for the specific anatomical location of the lesion (jaw bones) and the timing of the disease’s occurrence and regression is not yet deciphered. However, the most accepted hypothesis explaining both aspects proposes a putative link to definitive tooth eruption (18).

Owing to its benign, mostly regressive course and no findings in routine biology assays, cherubism remains poorly addressed in endocrine or rheumatology departments in terms of a diagnosis assessment and long-term medical follow-up. Thus, despite several lethal cases, surgeons and dentists have established the management protocols, largely aimed at functional and aesthetic outcomes. Major concerns are the management of dental sequelae and orthodontic issues. Moreover, the natural history of cherubism implies spontaneous regression, which means that surgical management is discussed only in very aggressive cases (orbital involvement, impact on tooth eruption, nasal obstruction, glossoptosis (19, 20)). Conservative curettage is the most commonly performed surgical technique; however, surgery remains controversial and may result in irreversible lesions (21).

Although the disease was first described in the 1930s, a suggestion for drug management was not proposed until 2000 (22). Furthermore, despite impressive advances in the genetic and molecular understanding of cherubism, no drug has yet been tested in a clinical trial. In several cases throughout the last decades, various off-label drugs have been tried but with differences in treatment duration, protocol and heterogeneity in the identification of the specific effect of the drug. Calcitonin was the first drug reported; however, its efficacy in cherubism lesions remains unclear. There is still no agreed-upon recommendation for drug management of cherubism, probably due to the low number of cases and the heterogeneity of presentations. To our knowledge, there is no systematic review aimed at compiling the different pharmacologic agents given to cherubism patients and their effectiveness. Therefore, the aim of this systematic review was to answer the following question: Is there an evidence-based effective treatment for cherubism?

## Methods

### Protocol and registration

This systematic review was conducted following the guidelines of the Preferred Reporting Items for Systematic Reviews and Meta-analysis checklist (PRISMA) (23). The systematic review protocol was registered at the International Prospective Register of Systematic Reviews (PROSPERO) under number CRD42022351044.

### Eligibility criteria

The studies selected in this review involved patients, either children or adults, with cherubism and for which there was pharmacological management of the disease. The cherubism diagnosis was defined based on clinical, radiological and sometimes histological and genetical analyses (as previously summarized in (4)).

Overall, the inclusion criteria were based on the PICOS methodology (24): Population (P): cherubism subjects; Exposure (I): pharmacological management; Comparison (C): different therapies, placebo, no therapy or no comparison; Outcome (O): effective treatment; Study design (S): clinical trials, randomized studies, observational studies, case reports and case-series. Included studies needed to describe details on drug delivery and whether these treatments affected the disease evolution. No publication period or time restriction was applied. Language was limited to English and French. The following exclusion criteria were applied: 1) Studies in which only surgical management was suggested; 2) Studies on animal models; 3) Studies with no treatment; and 4) Literature reviews and personal opinions.

### Information sources and search strategy

Literature searches were performed using PubMed (including Medline), EMBASE, Web of Science, and Scopus databases. Electronic database searches were conducted from their starting coverage date through February 1, 2022. More information on the search strategy is provided in **Appendix 1** (which can be found online).

All references were managed and the duplicated hits were removed by using reference manager software (EndNote X7^®^ Basic-Thomson Reuters, New York, USA).

### Selection process

Article selection followed a two-phase process. In phase 1, two reviewers (A.E.C. & P.-E.C.) independently screened all published papers meeting the inclusion criteria in the electronic databases, using titles and abstracts. In phase 2, the same two authors independently evaluated the full text of each paper, applied the eligibility criteria, collected key information from the selected studies, and crosschecked the information. The final selection was based solely on full-text assessment of the studies. Discrepancy in paper selection led to discussion until mutual agreement. When disagreement occurred, a third author (A.L.P) was involved to make a final decision about whether to include or exclude a study.

### Data collection and data items

The data collected consisted of study characteristics (authors, year of publication, country, design), population characteristics (sample size, age of participants, demographic features), methods, drug (type, dose, posology, route of administration) and outcome characteristics on cherubism (findings, timespan, follow-up and main conclusions).

### Study risk of bias assessment

The selected studies were evaluated using the Joanna Briggs Institute’s critical appraisal tools to assess risk of bias. The answer could be ‘yes’, ‘unclear’, ‘no’, or ‘not applicable’. Two reviewers (A.E.C. & P.-E.C.) independently classified the quality of each included study. In case of discrepancy, the two reviewers conciliated. Following these ratings, the risk of bias was categorized as high, if one or more criteria were not met; low, if all criteria were met; or unclear, if one or more criteria were not rigorously described (23). Figures of the quality assessment of all studies were generated using Review Manager software (RevMan v.5.3, The Nordic Cochrane Center, Copenhagen, Denmark).

### Effect measures and synthesis methods

*A priori* the following analyses were considered, and applied where appropriate: 1) Any effect on disease progression, e.g. as measured by mean and standard deviation; 2) Quantitative synthesis, including a meta-analysis (using RevMan 5.3); 3) Tests of heterogeneity using the Cochran Q test and I^2^ statistics; 4) A fixed or random effect model based on the heterogeneity values detected, where a value greater than 50% may be considered as an indicator of substantial heterogeneity between studies.

### Risk of bias across studies and reporting bias assessment

The risk of bias across studies was assessed as an overall risk which could influence a meta-analysis. Methodological and statistical heterogeneity were evaluated by comparing the variability in study design and the risk of bias.

When the required data were not complete, the reviewers (A.E.C. & P.-E.C.) attempted to contact the study authors to obtain specific unpublished information. Three attempts were made in a 30-day period, by email to the first, second and last authors.

### Certainty assessment

A summary of the overall strength of evidence available was presented using “Grading of Recommendations Assessment, Development and Evaluation” (GRADE) Summary of Findings (SoF) tables, using GRADE pro software (25).

### Data availability statement

All data, materials and methods which support the results can be found in the article or the appendices.

## Results

### Study selection

**Figure 1:**
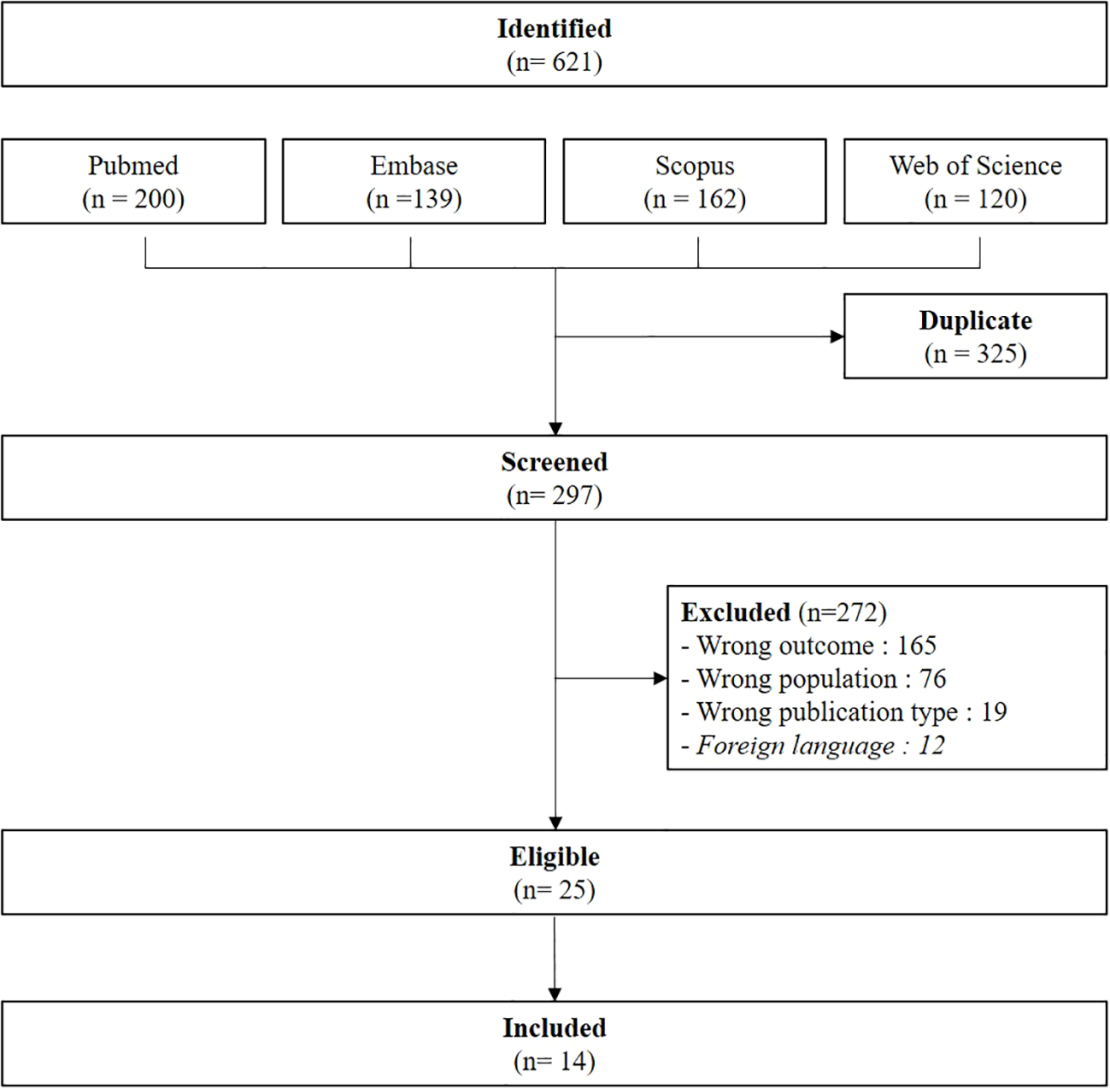
Flowchart of the literature search and selection criteria.

The initial database search identified 621 articles. After eliminating duplicate hits, 297 articles remained of which 272 were excluded after title and abstract review, leaving 25 articles for phase 2. During phase 2, nine more articles were excluded (reasons for exclusion can be found in **Appendix 2**), leaving 14 articles for qualitative synthesis. A flowchart of the process of identification and article inclusion and exclusion are shown in **Figure 1**.

### Study characteristics

The 14 included studies were mostly case reports. The studies were conducted in Brazil (26), Cyprus (27), France (28), Finland (29), Israel (30), Italy (31), Japan (32), the Netherlands (33), Russia (34), Turkey (35), United Kingdom (36, 37), United States of America (38, 39). All studies were in English. The studies reported administration of various treatment options, including calcitonin, denosumab, oral bisphosphonates, adalimumab, tacrolimus and imatinib. The descriptive characteristics of the included studies are summarized in **Table 1**.

**Table 1:** Types of papers.

Article number	Reference	Type of article	Journal scope	Publication date	Study location	No. of cases	Authors’ specialty	Tested drug
**Art.1**	Bar Droma et al. (30)	Case report	Oral/MF surgery	2020	Israel	2	Pediatrics, oncology, MF surgery	Denosumab
**Art.2**	Bradley et al. (36)	Case report	Oral/MF surgery	2020	England	1	MF surgery	Alendronate
**Art.3**	Dateki et al. (32)	Case report	Pediatric endocrinology	2020	Japan	1	Pediatrics	Denosumab
**Art.4**	de Lange et al. (33)	Case report	Oral/MF surgery	2007	Netherlands	1	MF surgery	Calcitonin
**Art.5**	Etoz et al. (35)	Case report	Dentistry	2011	Turkey	1	Dentistry	Calcitonin
**Art.6**	Fernandes Gomes et al. (26)	Case report	Dentistry	2010	Brazil	1	Dentistry	Calcitonin
**Art.7**	Hero et al. (29)	Case report	Bone metabolism	2013	Finland	2	Pediatrics, MF surgery, radiology	Adalimumab
**Art.8**	Kadlub et al. (28)	Case report	Bone metabolism	2015	France	1	Oral/MF surgery, dentistry, genetics, pathology, biology	Tacrolimus
**Art.9**	Kugushev et al. (34)	Case report	Tumor research	2018	Russia	1	Oral/MF surgery	Denosumab
**Art.10**	Lannon et al. (37)	Case report	Plastic surgery	2001	Ireland	1	Plastic surgery	Calcitonin
**Art.11**	Pagnini et al. (31)	Letter	Rheumatology	2011	Italy	1	Pediatric rheumatology	Alendronate/Adalimumab
**Art.12**	Ricalde et al. (39)	Case report	Oral/MF surgery	2019	USA	3	MF surgery, pediatric oncology, dentistry	Imatinib
**Art.13**	Upfill-Brown et al. (38)	Original article	Bone metabolism	2019	USA	1	Orthopedics, MF surgery, oncology, pediatrics	Denosumab
**Art.14**	Zoe et al. (27)	Case report and narrative review	Oral/MF surgery	2021	Cyprus	1	Dentistry	Calcitonin

### Patient characteristics

**Figure 2:**
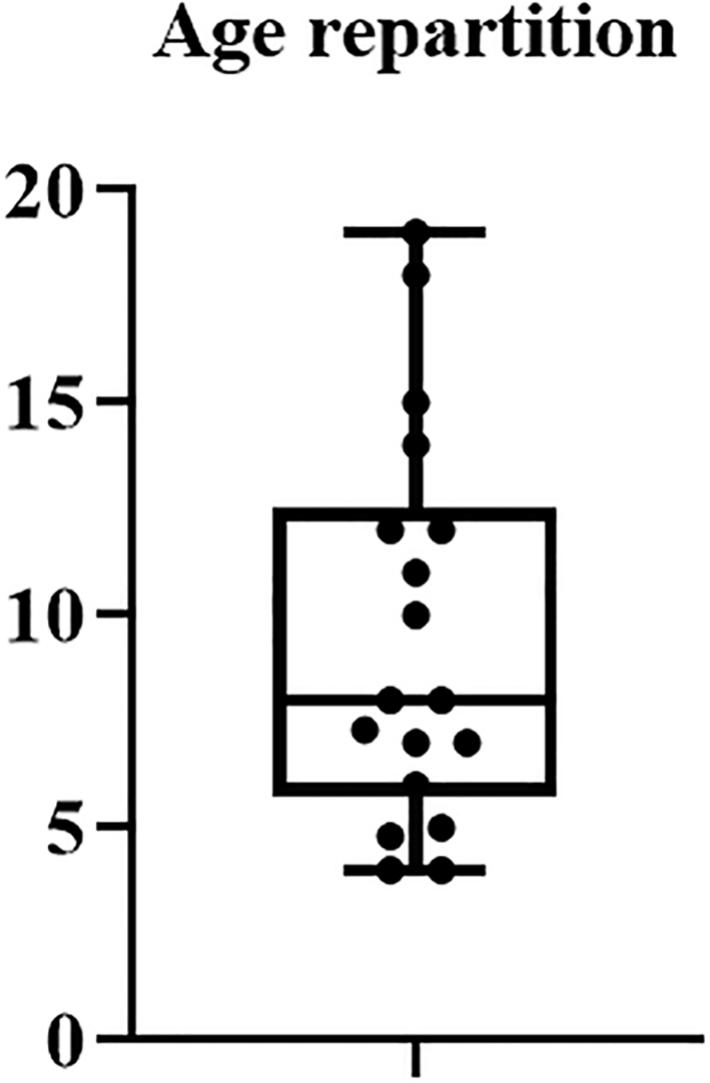
Patient distribution by age.

In the 14 included studies the sample sizes ranged from one (26–28, 31–33, 35–38, 40) to three (39) patients, with a total of 18 subjects with cherubism. Seven patients (38.9%) were female. The mean age of the subjects was 9.6 years, ranging from 4 to 19 years (**Figure 2**). When stated, the age at diagnosis ranged from 2 to 6 years, with a mean age of 4 years. The cherubism patients were from different ethnic origins (Caucasian (7 patients - 38,9%), Black (3 patients – 16,7%) or Asian (2 patients – 11,1%)), even if for 6 of them nothing was specified. Two patients were reported to be twins: one was a fraternal twin (with the brother having minor signs of cherubism) (30) and for the other the twin-type was not specified (31). Of the 18 patients, six (33.3%) were treated with denosumab, seven (38.9%) with various immune/inflammatory modulating drugs, and five (27.8%) with calcitonin. **Table 2** summarizes the patient demographics.

**Table 2:** Patient demographics.

	Age (civil)	Age at diagnosis	Sex	Ethnicity	Family history	Twinship
Bar Droma et al. (30)	19	nm	F	Caucasian	Yes	No
Bar Droma et al. (30)	15	nm	F	Caucasian	Yes	Y
Bradley et al. (36)	13	6	M	Black	nm	nm
Dateki et al. (32)	10	4.5	M	Asian	Yes	No
de Lange et al. (33)	11	nm	M	Caucasian	nm	nm
Etoz et al. (35)	14	4	M	Indian	nm	nm
Fernandes Gomes et al. (26)	18	nm	F	Caucasian	nm	nm
Hero et al. (29)	7.3	4.4	M	nm	nm	nm
Hero et al. (29)	4.8	4.5	F	nm	nm	nm
Kadlub et al. (28)	4	2	M	nm	Yes	nm
Kugushev et al. (34)	9	~ 6	M	Caucasian	nm	nm
Lannon et al. (37)	7	nm	M	Caucasian	nm	nm
Pagnini et al. (31)	5	3	F	nm	Yes	Y
Ricalde et al. (39)	8	6	M	Caucasian	nm	nm
Ricalde et al. (39)	6	3	M	Black	Yes	nm
Ricalde et al. (39)	4	2	F	Black	Yes	nm
Upfill-Brown et al. (38)	12	5	F	nm	nm	nm

nm, not mentioned.

In the 18 patients, diagnosis of cherubism was establish by clinical features; radiographic imaging, which allowed the cherubism severity to be graded; pathoanatomical analysis; and since 2001 (17) identification of the causative mutation, including genetic assessment of *SH3BP2* mutations. For 10 patients, pathognomonic bilateral cherubism lesions were reported. Radiographic images were provided for all 18 patients. However, the severity grade and the classification used were given for only two patients (28, 35). For seven patients (38.9%), a familial history of cherubism was mentioned. In the 18 patients, two were sisters (30) and two were cousins (39). We decided to include cherubism patients with or without genetic data. Thus, for nine patients, no genetic analysis of *SH3BP2* mutations was mentioned, and for one patient it was clearly stated that no genetic analysis was conducted because of the clear cherubism diagnosis (31). For eight patients, a genetic diagnosis was made, and the exact mutation was given for five patients (28, 29, 32); one study reported a mutation in *SH3BP2* exon 9 but did not give the specific mutation (33). Very few extraoral cherubism manifestations were mentioned such as exophthalmia (30, 31) and dysphagia (39). **Table 3** summarizes the cherubism diagnostic features of the patients.

**Table 3:** Cherubism diagnostic features.

Patient	Article	Age	Cherubism family history	Clinical diagnosis	CBCT before treatment	Severity grade	biopsy before treatment	Genetic analysis	SH3BP2 mutation
**1**	Bar Droma (30)	19	yes	Cherubism diagnosis already made	yes	nm	yes, few GMC	yes before	nm
**2**	Bar Droma (30)	15	yes (sister of 1)	Cherubism diagnosis already made	yes	nm	yes, numerous MGC	yes before	nm
**3**	Bradley (36)	12	nm	painless bilateral maxillary and mandibular swelling	yes	nm	No	nm	na
**4**	Dateki (32)	10	yes	Cherubism diagnosis already made	yes	nm	No	yes	p.Pro418 Arg
**5**	De Lange (33)	11	yes	bilateral maxillary and mandibular swelling	X-ray	nm	yes, MGC	yes	mutation in exon 9
**6**	Etoz (35)	14	nm	painless bilateral maxillary and mandibular swelling	yes	Grade I (Motamedi + Seward & Hankey)	yes, MGC	nm	na
**7**	Fernandes Gomez (26)	18	nm	bilateral maxillary and mandibular enlargement	yes	nm	yes, MGC	nm	na
**8**	Hero (29)	7,3	nm	painless bilateral maxillary and mandibular swelling	yes	nm	yes	yes	p.Pro418 Leu
**9**	Hero (29)	4,8	nm	expansion of the mandibular symphysis	yes	nm	yes	yes	p.Pro418 His
**10**	Kadlub (28)	4	yes	painless bilateral maxillary and mandibular swelling	yes	Grade IV (Seward & Hankey)	yes, MGC	yes	p.Pro418 Arg
**11**	Kugushev (34)	8	nm	increase of the lower jaw	yes	nm	yes, MGC	nm	na
**12**	Lannon (37)	7	nm	profound mandibular hyperplasia	X-ray	nm	yes, MGC	nm	na
**13**	Pagnini (31)	5	yes	painful swelling of the cheeks	yes	nm	not mentioned	no genetic	na
**14**	Ricalde (39)	6	nm	left mandibular swelling	yes	nm	yes, MGC	nm	na
**15**	Ricalde (39)	8	yes	jaw swelling	yes	nm	yes, MGC	nm	na
**16**	Ricalde (39)	4	yes (cousin to 15)	bilateral expansion of the jaw	yes	nm	yes, MGC	nm	na
**17**	Upfill-Brown (38)	12	na	jaw enlargement	yes	nm	nm	nm	na
**18**	Zoe (27)	7	na	bone expansion of the mandibular body	yes	nm	nm	nm	na

GMC, multinucleated giant cells; nm, non-mentioned; na, not applicable.

### Risk of bias in the studies

**Figure 3:**
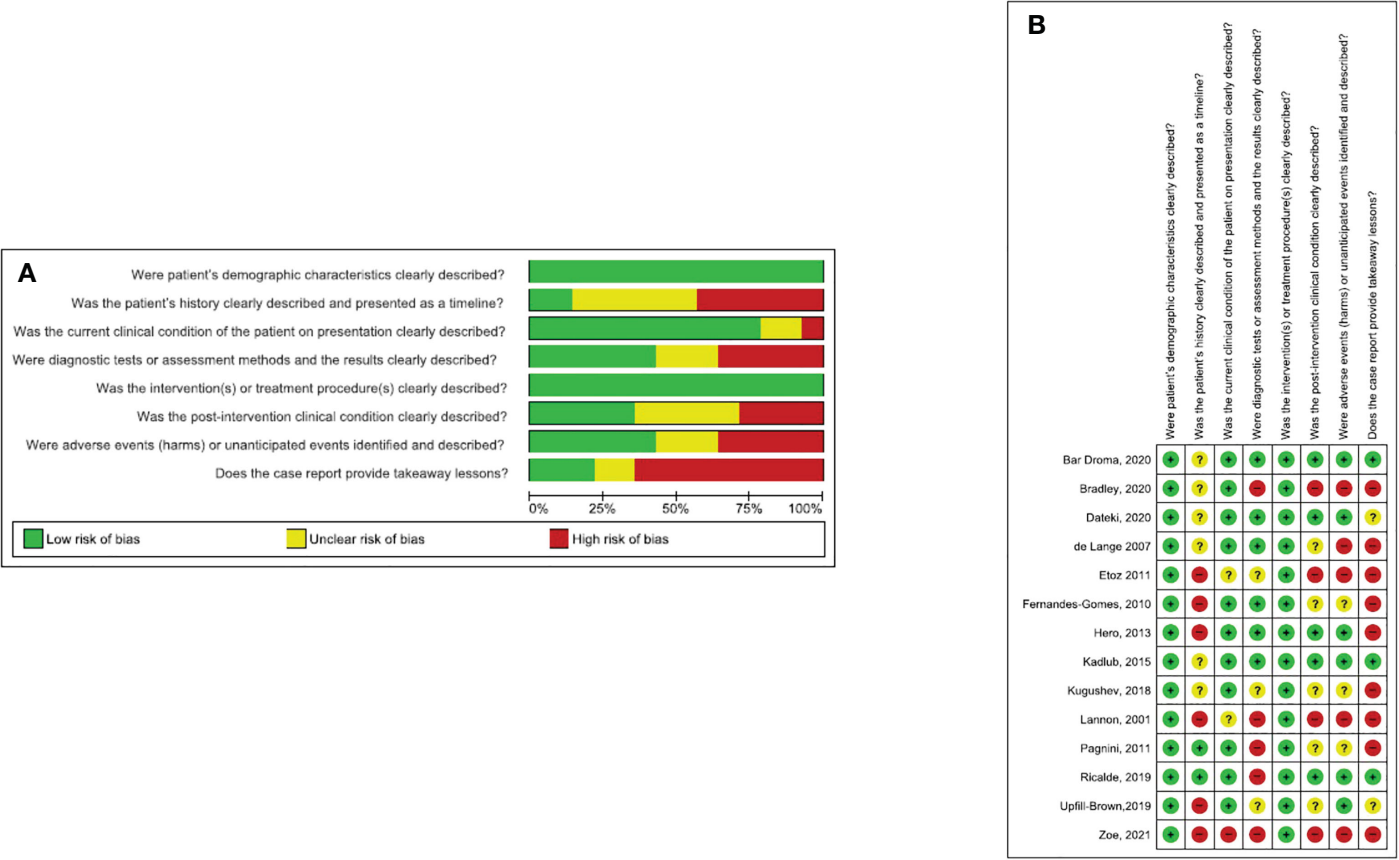
Risk of bias. **(A)** Risk of bias graph: review authors’ judgments about each risk of bias items presented as percentages across all included studies. **(B)** Risk of bias summary: review authors’ judgments about each risk of bias item for each included study.

Risk of bias was heterogeneous among the studies. Using JBI critical appraisal tools, five studies (28–30, 32, 39) were classified as low risk of bias, four (26, 31, 33, 38) as unclear risk of bias and five (27, 35–37, 40) as high risk of bias. No study fulfilled all the methodological criteria. However, as expected of case reports, all 14 articles presented clear descriptions of the patient characteristics and the treatment provided. The sources of high risk of bias included incomplete reporting of the patient history, lack of details about the method of diagnosis, incomplete post-intervention clinical condition of the patient, lack of reporting of adverse events, and absence of takeaway lessons. The complete item list is presented in **Figure 3** and **Appendix 3**.

### Results of syntheses

A meta-analysis was not performed because of insufficient data for statistical pooling related to the type of study included; all were case reports, with a maximum of 3 patients.

A summary of the data collected concerning the treatments given to the 18 cherubism patients is presented in **Table 4**. Interestingly, the number of drugs administered is relatively low (calcitonin, denosumab, bisphosphonates, anti-TNFα, tacrolimus, imatinib) and can be divided in three categories: anti-resorptive treatment, immunomodulating treatment, and calcitonin. If we consider those three categories individually, it is worth noting that for the same drug or class of drug, neither the dose, the cumulative dose, the mandatory co-prescription? (e.g. Vitamin D and Calcium for denosumab) or even the administration route was consistent among studies.

**Table 4:** Outcomes on drug efficacy.

	Ref.	Age at drug initiation (years)	Tested drug	Dose	Administration	Frequency	Surgery	Co-prescription	Reason for initiation	Reference for treatment choice	pre-treatment set	Reason for interruption	Major adverse effects	Ethic discussion and committee	Authors conclusion
**Anti-resorptive**	Bar Droma et al. (30)	15	Denosumab	120 mg	subcutaneous	10 doses: D1, D8 D15, D28 + 1/28-d	Before	Oral calcium and vitamin D	severity	Central giant cell granuloma treatment protocol	dental extraction, Cone beam, biology	scheduled	NO	Yes	Bone become denser
		19							severity			scheduled	NO	Yes	
	Dateki et al. (32)	10	Denosumab	120 mg	subcutaneous	8 doses: D1, D7, D28, + 1/28-d	NA	None	Extent of lesions. Impossibility of surgery	Central giant cell granuloma treatment protocol	NA	scheduled	Severe Hypocalcemia at 1^st^ injection and failure to thrive M2 to M11	Yes	Suppression of the expansion of the osteolytic lesion and dramatic ossification
	Upfill-Brown et al. (38)	12	Denosumab	120 then 60 mg	subcutaneous	14 doses: D1 (120) then 60,	NA	Aminocaproïc acid (hemorrhage)	Disease progression pain, gingival hemorrhage	Off study, off label	NA	scheduled	Severe adapted-PTH Hypocalcemia at 1^st^ injection	NA	Excellent radiographic response with increased sclerosis
								opioid analgesics							
	Kugushev et al. (34)	9	Denosumab after oral-bisphosphonate	120 mg	subcutaneous	7 doses: D1, D8, D15 +1/28-d	Impossible	Oral calcium 500 mg/d Vitamin D 500 IU/d	severity, impossible surgery, exophthalmos	Metastatic giant cells tumor of the bone	Parathyroid hormone, Calcium,Phosphate, vitamin D	scheduled	Hypophosphatemia at M3 (no interruption)	Yes	Increased jaw bone density
															**Unmodified skeletal age?**
	Bradley et al. (36)	13	Alendronate	70 mg	oral	78 doses (26 doses 1/week 6 months then hold for 18 months then 52doses 1/week 12 months)	Maxillectomy just before treatment initiation	None and no prescription recommendations	Rapid evolution. Tumor volume with side effects: dysphagia, loss of weight, dyspnea, stress failure to thrive	Empirically, off study off label	NA	Not really scheduled. Inobservance.	NA	NA	No more progression New sequence after 24 months off treatment because of new lesions
**Various immuno-modulating/inflammatory drugs**	Pagnini et al. (31)	5	Alendronate + Adalimumab	35 mg (ALN)	oral (ALN)	ALN: 36 doses 1/week 9 months	NA	NA	evolution and family request	Empirical and mouse data	NA	At M9 (1^st^ follow-up) for slight progression	NA	Yes	Ineffectiveness of the treatment
				24mg/m² (Admab)	subcutaneous (Admab)	Admab: 18 doses (1 per 2 weeks)									
	Hero et al. (29)	7	Adalimumab	40 mg	subcutaneous	54 doses (1 per 2 weeks 27 months)	Y	NA	dental severity only	Mouse data	Biological extensive set: Ca, Phosphate, PTH, and bone biomarkers	progression, need for surgery	non severe recurrent respiratory tract infections (suspension)	Yes	Progression, with necessity for surgery during the treatment
		4	Adalimumab	40 mg	subcutaneous	62 doses (1 per 2 weeks, 31 months)	Y	NA	dental severity only	Mouse data		scheduled	pneumonia (suspension)	Yes	No progression
	Kadlub et al. (28)	4	Tacrolimus	0.15 mg/kg/j	oral	2 doses	Y	NA	severity (sleep apnea). Inefficiency of surgery with recurrent nasal obstruction	Aggressiveness marker	liver markers. No bone markers,	scheduled	NA	Yes	Efficiency based on the before/after comparison in markers lequels?
	Ricalde et al. (39)	8	Imatinib	200 mg (300mg/m²)	oral	> 180 doses (6 months) Then, inobservance. Loss to follow-up 1-y	Y	ibuprofene, paracetamol	Pain, dysphagia with failure to thrive and hemorrhage. Family request and social issues.	Off study, off label	NA	according to clinical efficiency	NA	NA	Clinical regression with – 75% lesion size but histologically: same lesions
		6	Imatinib	300 mg (300mg/m²)		304 doses (10 months)	Y	iron supplementation (anemia)and setron	severity (sleep apnea, hemorrhage with anemia)	Off study, off label	NA	according to clinical efficiency	nausea	NA	At M10 Decrease of 22% of lesion size
		4	Imatinib	200 mg (300mg/m²)		NA	Y	setron	Relative of another case. Failure of surgery	Off study, off label	NA	according to clinical efficiency	nausea	NA	Decrease in lesion size (- 65%) at M10
**Calcitonine**	Etoz et al. (35)	14	Calcitonine	200 IU	intra-nasal spray	910 doses:1/d during 30 months	Y	NA	exophthalmos with minor oral form	Off study, off label	NA	according to clinical efficiency	NA	NA	Clinical improvement at M30
	Fernandes Gomes et al. (26)	18	Calcitonine	200 IU	intra-nasal spray	365 doses (1/d, 1 year)	Y with auto-graft	NA	“aggressive cherubism” with no extra-oral damage	NA	Calcium, Phosphate, parathyroid hormone (PTH), calcitonine, ALP	scheduled	NA	NA	Improvement
	de Lange et al. (33)	11	Calcitonine	200 IU	intra-nasal spray	NA	Y	NA	Pain	Central giant cell granuloma treatment protocol	PTH calcium	scheduled	(unevaluated Irradiation with scans every 3 months)	NA	Initial regression after 15 months
						(1 year)									
	Lannon et al. (37)	7	Calcitonine	100 UI	subcutaneous	6 months	Y	NA	Dysphagia	Central giant cell granuloma treatment protocol	NA	NA	NA	NA	No improvement. Need for surgery
	Zoe et al. (27)	7	Calcitonine	200 IU	intra-nasal spray	1 100 doses	Y	NA	NA	NA	PTH	NA	NA	NA	Marked resolution of the lesion
						1/d during 30 months									

NA, not applicable; Y, yes; PTH, parathyroid hormone.

In most cases, the drug treatment was administrated as part of a therapeutic strategy to prepare for eventual surgery (**Table 4**). The rationale for the choice of the drug therapy was not always clearly stated. In four articles (30, 32, 33, 37), denosumab and calcitonin were chosen because they are used in the treatment of central giant cell granuloma. Only two treatments were based on previous data (28). In one report, the treatment was off study and off label (39). Initiation of treatment was usually associated with the severity of the cherubism lesion. Plans for administration of the treatment were discussed and validated by a multidisciplinary team and/or an ethics committee for nine patients. Seven patients were subjected to light to thorough pre-treatment analyses (blood markers, etc.) (**Table 4**). Adverse effects such as nausea, hypophosphatemia, hypocalcemia (sometimes severe (32)) were reported in eight patients. The choice of the duration and cessation of treatment was not systematically explained.

The clinical outcomes of the treatments are also a source of heterogeneity (**Table 4**). Only for two patients (31, 37) did the authors clearly state the treatment was ineffective (anti-TNFα and calcitonin). For two more patients the treatments were said to be insufficient, as there was recurrence of the cherubism lesions (29). Regarding the age of the patients at treatment initiation, 10 of the patients were under 10 years old and four were above 13.

### Reporting biases

It was not established any risk of bias due to missing results (arising from reporting biases) in this study.

### Certainty of evidence

The overall quality of evidence identified using GRADE SoF tables was assessed as being very low (**Appendix 4**), because of high inconsistency due to the different therapies, unclear outcomes, small sample size, and study designs classified as observational studies.

## Discussion

This systematic review, which included 14 studies, aimed to determine if an effective treatment for cherubism has been identified. The significant heterogeneity in the data reported in the studies and the type of included studies (case reports) precluded a meta-analysis. Moreover, the heterogeneity made synthesis of the data challenging. The number of drugs given to cherubism patients was relatively low (calcitonin, denosumab, bisphosphonates, anti-TNFα, tacrolimus, imatinib) and may be divided in three categories: anti-resorptive treatment, immunomodulating treatment, and calcitonin. Overall, the data summarized here make any conclusion regarding drug efficacy quite uncertain. More standardized and rigorous studies are needed. These will probably require participation of multiple centers worldwide, as cherubism is a rare condition. To that end, this systemic review allowed us to define a new checklist of items that should be included in any such studies (**Appendix 4**).

### Study limitations

Cherubism is a rare bone disease, with slightly more than 500 case reports in the literature (1). The rarity of the disease explains the low number of papers that were included in the present systematic review. It is noteworthy that even though most of the included studies were case reports, there was great heterogeneity not only in the cases reported and in the treatments administered, but also in the way the cases were reported, which might reflect the diversity the journals where they were published. As all 14 articles included in this systematic review are case reports, the overall quality of evidence of the studies was categorized as very low, according to the GRADE criteria (25). The level of evidence was downgraded due to limitations in study designs, imprecision, and inconsistency because of evident heterogeneity across the studies. Another strong limitation is that the absence of clear expected outcomes at the end of the treatments on cherubism progression greatly challenged our efforts to evaluate the clinical implications that can be drawn from those studies. That deficiency and a lack of standardization for reporting renders the findings inconclusive, even the results of the five patients who received denosumab treatment. In addition, it’s not clear that all the cases reported as cherubism were in fact true cases of cherubism. Indeed, the pathognomonic features of cherubism are bilateral symmetric lesion of osteolysis without pain; however, some cases reported pain and non-symmetric lesions. This increased the heterogeneity of the case reports systematically reviewed here.

### Patient characteristics

In the 14 papers we reviewed, the characteristics of the patients were clearly described (**Figure 2**), and this revealed substantial variation among the patients in terms of sex, cherubism severity, and age at treatment initiation, thereby limiting comparisons. However, the disease natural history of every patient was not always clear, e.g., whether the cherubism was a sporadic or familial form was not systematically stated; similarly, the age at diagnosis. The diagnosis of cherubism is based on clinical features, radiographic images and histological features. If the first two were clearly given, the histological features were not always made explicit. The last piece of evidence allowing the establishment of a definitive cherubism diagnosis is identification of a mutation affecting the *SH3BP2* gene (17). It is understandable that sequencing facilities might not be available in all care settings, but each report should contain a statement about whether a mutational analysis was done or not. Our analysis underscores the need for definition of a standardized set of basic information to be included in all cherubism reports going forward (see **Appendix 4**). We recommend that basic biologic assessment be done and reported before, during and after the treatment, and refer to the age and sex appropriate norms for this pediatric population.

### Choice of treatment

Our analysis revealed three categories of treatments that were given to cherubism patients throughout the past 20 years or so. After Ueki’s team demonstrated that TNF-α was central in the initiation and maintenance of the cherubism phenotype in mice (41), it seemed appropriate to try to treat cherubism patients with an anti-TNFα drug such as adalimumab, as did Hero and colleagues (29) or Pagnini (31). However, the clinical outcomes from these attempts were less than convincing and the therapies were discontinued. Severe cherubism is characterized by re-localization of NFATc1 into the nuclei of those giant cells (20, 28). These observations led our team (28) to treat a patient exhibiting severe cherubism with tacrolimus in order to prevent NFATc1 translocation. This treatment appeared to stop the progression of the cherubism, but it did not resolve the disease.

Cherubism and central giant cell granulomas share some features such as fibrotic lesions containing multinucleated cells as well as their localization in bone, specifically in jaw bones (42). Accordingly, therapies used to treat central giant cell granulomas would seem to be natural therapeutic candidates for cherubism — calcitonin and denosumab might be examples. However, based on our present analysis, calcitonin may not be an effective treatment for cherubism, and this appears to be the case for many other diseases in which calcitonin has been tried. Indeed, calcitonin’s use in bone diseases is declining as new and more effective drugs are being developed (43). Nonetheless, nasal administration of calcitonin appeared as an interesting alternative route, especially for young patients (20, 28). Concerning denosumab, one can roundly question its use in cherubism patients: the drug has not been authorized for use in children; moreover, the dose used (120 mg) is the same as that given to adult patients for malignant diseases. Clearly, off-label off-study treatment of cherubism patients remains a concern. Gaining insight into the pathophysiology of cherubism is imperative to be able to offer an effective and evidence-based treatment to patients.

Related to this improvised approach, our analysis revealed that an expert multidisciplinary team or an ethics committee was involved in the treatment decision for only 9 of the 18 patients. This raises concerns about both the way treatment decisions are made and, more broadly, the way cherubism patients are cared for, as very few pediatricians, bone specialists or endocrinologists were involved or mentioned.

### Choice of initiation of treatment

A critical piece of information missing from most of these studies is the severity grade of cherubism, as it was given only for two patients. The evolution of cherubism is not well understood; however, several severity classifications have been elaborated through the years, the oldest and simplest being that by Seward and Hankey (6) with three grades, and the most complex having six severity grades, some with up to five subclasses as proposed by Raposo-Amaral (7) or Motamedi (8). The most recent classification defines a new severity grade (the seventh), when cherubism is associated with other syndromes (9). The severity grade should also be considered in discussions about whether and when to initiate a treatment. Although the mechanism underlying disease resolution remains unknown, the expectation of eventual resolution raises questions concerning the age at which treatment initiation might be started. In our analysis, among 18 patients, four were above 13 years old, and two were young adults. Obviously, without a clear severity grade associated with specific age groups, it is difficult to judge the opportuneness of the described treatment. This highlights the importance of the severity grading (and the choice of classification) especially in the context of patient age. Because the latter is an important factor in the disease course, it raises the question of how natural resolution versus the effect of a drug can be distinguished.

### Drug administration, follow-up, adverse effects

The desire to repurpose various drugs to treat cherubism is highly understandable. However, when choosing a drug, its dose, and its way of administration, the clinician should be mindful that it is mostly children who will be treated. Decisions on ‘new’ drug treatments should be informed by the results of previously published reports. During follow-up of the effect of the treatment, imaging might be necessary. Again, the concerned population (children) should always be kept in mind, and limiting excessive radiation exposure should always guide the choice of the type of imaging. The anticipated adverse effects of drugs should be monitored, for example hypocalcemia in the case of denosumab (32, 38). The duration of the treatment was not always made explicit in the included articles, and for some, it was even suggested that the treatment was still ongoing when the paper was published. This information is essential and should be clearly stated, and follow-up until the end of the treatment should be included. Furthermore, any adverse effects, even well after treatment discontinuation, should be mentioned and bone markers should have been tested before, during and after the cessation of the treatment, but generally were not.

### Outcomes

While the aim of the treatment was not always clearly stated, all 14 case reports sought to improve the condition of the cherubism patients. Most of the outcomes that were mentioned focused on the jaw-bone aspect, and some also concerned the lesions themselves, with reduction of the numbers of multinucleated giant cells or their activation. However, neither the outcomes nor their evaluation was standardized, and this lack of consistency should be improved.

Treatment of cherubism patients with calcitonin had various effects, from no improvement (37) to reported clear improvement (27). If we assume calcitonin has some efficacy, differences in the duration of treatment, the dose and also patient age and probably cherubism severity might explain the discrepancy.

A striking effect of treatment with denosumab is that jaw-bone sclerosis was observed irrespective of the dose, the duration, the age and probably the cherubism severity (for example, see the images in (34)). But, how satisfied should we be with transforming the soap-bubble like cavities of the jaw bone into sclerotic bone, and does this really improve the patient’s condition and the disease progression? This is highly questionable, especially in a mainly spontaneously regressive disease. In addition, apart from the known side-effects of denosumab, its long-term effects are poorly reported and the high doses given to children remain a concern. External expert oversight, even in studies granted ethical approval, needs to be improved. What is now clearly missing is clinical information about the state of the jaw bones of those patients long after the treatment. One study stated that the serum level of C-terminal telopeptide increased (30) after the discontinuation of the treatment (i.e., some bone renewal had resumed).

Alendronate alone was reported to be associated with a cessation of the progression of the lesions, but the study involved only a single patient, 13 years old (36). When combined with an anti-TNF-α, in another study, the authors concluded that the treatment was ineffective (31). Anti-TNF-α alone was also declared to be an ineffective treatment (29).

Tacrolimus treatment given to one of our own patients, although with a peculiar dosing, appeared to be effective in reducing measurable intermediary outcomes related to cherubism activity (such as a decreased number of TRAP-positive multinucleated giant cells, the nuclear location of NFATc1, and the RANKL/OPG ratio of histological samples). In this unique patient with severe cherubism, we observed a cessation of both lesion progression and osteolysis after tacrolimus administration (28). This treatment, supported by evidence of NFATc1 pathway involvement in cherubism and with measurable outcomes, paves the path to stronger studies targeting both osteoclasts and immune cells, as tacrolimus does (28).

Clinical outcomes were satisfying with imatinib, a tyrosine kinase inhibitor, as the volume of the lesions decreased (39). Interestingly, this off-study and off-label treatment efficient to decrease the volume of the patients’ lesions did not have any effect on the cherubism mouse (44). However, Yoshimoto and colleagues demonstrated that treating with a very new second-generation SYK inhibitor (Entospletinib) rescued the cherubism phenotype (45). However, mainly adult patients suffering for malignant hemopathies (such as acute myeloid or lymphoblastic leukemia), were so far included in clinical trials (46)(see clinicaltrials.gov for details). Only few cellular tests have been conducted with this molecule in cells from infant acute lymphoblastic leukemia (47, 48). So Entospletinib could pave the way to a new approach incherubism therapy, however further studies are needed in this pediatric population.

### Checklist for conducting a case-report on therapy in a cherubism patient

Our analysis of the 14 included articles in the present systematic review prompted us to suggest a checklist for anticipating any cherubism treatment and helping to standardize its reporting, while waiting for new knowledge on cherubism pathogenesis or results from multicentric clinical trials. The items are listed in **Appendix 5**.

## Conclusion

Based on the relatively few drug therapies administered to a total of only 18 patients (calcitonin, denosumab, bisphosphonates, anti-TNFα, tacrolimus, imatinib), the question we sought to answer from this systematic review about treatment efficacy for cherubism could not be answered. The heterogeneity of the included articles in terms of patients, cherubism severity, treatment and outcomes prevented any clear conclusion. Indeed, this propelled us to suggest a standardized approach to testing and reporting of treatments until cherubism pathogenesis is better understood and thus able to provide a stronger footing for a rational and effective cherubism therapy.

## Data availability statement

The original contributions presented in the study are included in the article/**Supplementary Material.** Further inquiries can be directed to the corresponding authors.

## Author contributions

All authors have made substantial contributions: AC and P-EC initiated and coordinated the research; P-EC and AC managed and analyzed data; MC-S, AP, and NK participated in the data interpretation; and P-EC, AP, and AC wrote the article. All authors reviewed the article. All authors contributed to the article and approved the submitted version.
