# Supplementary material for: Pharmacological management of cherubism: A systematic review
Source: Front Endocrinol (Lausanne). 2023 Mar 14;14:1104025. doi: 10.3389/fendo.2023.1104025 (PMC10044089; doi:10.3389/fendo.2023.1104025)
Supplement: Supplementary file 1 [file DataSheet_1.docx]

**Appendix 1 : Database search strategy (May 2022)**

| Database | Search |
| --- | --- |
| PubMed | Cherubism [Mesh terms] OR Cherubism OR “Familial Fibrous Dysplasia of Jaw” OR “Familial Multilocular Cystic Disease of the Jaws” OR “Familial Benign Giant-Cell Tumor of the Jaw”  AND  Treatment OR “drug therapy” [Mesh Terms] OR “drug therapy” OR therapeutics [Mesh Terms] OR "pharmacological management" OR therapy |
| Scopus | TITTLE-ABS-KEY (Cherubism OR “Familial Fibrous Dysplasia of Jaw” OR “Familial Multilocular Cystic Disease of the Jaws” OR “Familial Benign Giant-Cell Tumor of the Jaw” AND Treatment OR “drug therapy” OR "pharmacological management" OR therapy) |
| Web of Science | (Cherubism OR “Familial Fibrous Dysplasia of Jaw” OR “Familial Multilocular Cystic Disease of the Jaws” OR “Familial Benign Giant-Cell Tumor of the Jaw” AND Treatment OR “drug therapy” OR "pharmacological management" OR therapy) |
| Embase | (Cherubism OR “Familial Fibrous Dysplasia of Jaw” OR “Familial Multilocular Cystic Disease of the Jaws” OR “Familial Benign Giant-Cell Tumor of the Jaw” AND Treatment OR “drug therapy” OR "pharmacological management" OR therapy) |
