# Supplementary material for: Pharmacological management of cherubism: A systematic review
Source: Front Endocrinol (Lausanne). 2023 Mar 14;14:1104025. doi: 10.3389/fendo.2023.1104025 (PMC10044089; doi:10.3389/fendo.2023.1104025)
Supplement: Supplementary file 2 [file DataSheet_2.docx]

**Appendix 2 : Articles excluded and the reasons for exclusion (n=11)**

| Author, Year (ref) | Reason for exclusion |
| --- | --- |
| Boot et al., 2007 [46] | **1** |
| Eiden et al., 2017 [47] | **2** |
| Elimairi et., 2019 [48] | **1** |
| Hart et al., 2000 [22] | **2** |
| Hart et al., 2000 [22] | **3** |
| Kawamura et al., 2019 [49] | **1** |
| Kugushev et al., 2019 [50] | **2** |
| Lopatin et al., 2018[51] | **2** |
| McMahon, 2007 [52] | **4** |
| Mozolovà et al., [53] | **2** |
| Schreuder et al., 2011 [54] | **1** |

Legend : 1. Abstract ; 2. Foreign language ; 3. Duplicate, 4. Review
