# Supplementary figures and images for: Pharmacological management of cherubism: A systematic review
Source: Front Endocrinol (Lausanne). 2023 Mar 14;14:1104025. doi: 10.3389/fendo.2023.1104025 (PMC10044089; doi:10.3389/fendo.2023.1104025)

**Appendix 3 : JBI Critical Appraisal Tools to assessed risk of bias in case-reports**

**
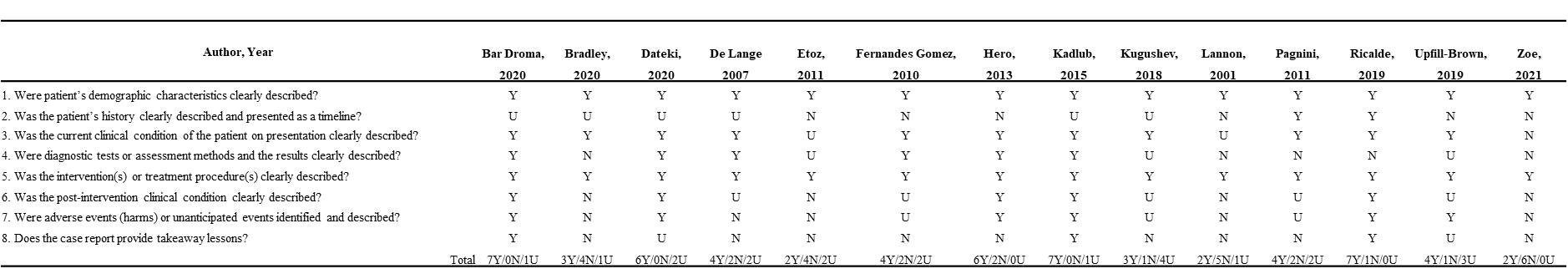
**

Supplement: Supplementary file 3 [file DataSheet_3.docx]
