# Supplementary material for: Pharmacological management of cherubism: A systematic review
Source: Front Endocrinol (Lausanne). 2023 Mar 14;14:1104025. doi: 10.3389/fendo.2023.1104025 (PMC10044089; doi:10.3389/fendo.2023.1104025)
Supplement: Supplementary file 4 [file DataSheet_4.docx]

**Appendix 4:** Summary of the overall strength of evidence using Grading of Recommendations Assessment, Development and Evaluation (GRADE).

| **Certainty assessment** | | | | | | | **Certainty** |
| --- | --- | --- | --- | --- | --- | --- | --- |
| **№ of studies** | **Study design** | **Risk of bias** | **Inconsistency** | **Indirectness** | **Imprecision** | **Other considerations** |  |
| 14 | observational studies | very serious^a^ | serious^b^ | not serious | serious^c^ | none | ⨁◯◯◯ Very low |

#### Explanations

a. Some studies presented a high risk of bias, mainly for the question: Does the case report provide takeaway lessons?

b. Even though we did not have enough data to perform a meta-analysis, we found high inconsistency in our results regarding outcomes that were not clearly defined and the application of different therapies.

c. The results lacked precision based on the low number of patients (18 included patients)
