# Supplementary material for: Pharmacological management of cherubism: A systematic review
Source: Front Endocrinol (Lausanne). 2023 Mar 14;14:1104025. doi: 10.3389/fendo.2023.1104025 (PMC10044089; doi:10.3389/fendo.2023.1104025)
Supplement: Supplementary file 5 [file DataSheet_5.docx]

**Appendix 5: Suggested Checklist for conducting a case-report on therapy in a Cherubism patient**

**NFATC-CC1: The Need For Anticipation in Treatments of Cherubism :**

This check-list aims to emphasize the steps of scientific reasoning that will allow you to formalize and optimize the use of data from your therapeutic practice in Cherubism patients.

| **PATIENT PROFILE** | **YES** | **NO** |
| --- | --- | --- |
| 1. Have you involved a physician known for his expertise in bone and pediatric fields (endocrinology, rheumatology) ? |  |  |
| If not, we recommend you do so or contact a member of one of the expert associations |  |  |
| 1. Was a genetic testing performed to assess cherubism? |  |  |
| 1. Have you performed an extensive set in order to explore other diagnosis options? |  |  |
| 1. Have you assessed severity with one of the 5 existing and validated tools? |  |  |
| 1. Does your patient suffer from extra-oral manifestations (orbital involvement, sleep apnea, dysphagia…)? |  |  |
| 1. Does your patient currently experience pain? |  |  |
| 1. What is the timespan between first symptoms and decision for medical therapy? |  |  |
| < 1 year |  |  |
| 1-4.5 years |  |  |
| > 4.5 years |  |  |
| 1. At this time, have you assessed bone age (Greulich & Pyle) and checked the growth curve? |  |  |
| 1. At this time, have you assessed the puberty stage (Tanner)? |  |  |
| 1. After a deeper assessment of your patient’s age, is puberty engaged? |  |  |
| If yes, have you anticipated techniques to distinguish spontaneous healing from drug effect? |  |  |
|  |  |  |
| **DRUG indication** | **YES** | **NO** |
| 1. Is your choice based on a previous recent study in another field (non-lethal disease)? |  |  |
| 1. If yes, did the authors reach a clear endpoint with patient improvement? |  |  |
| 1. If yes, did you find multiple examples with similar conclusions? |  |  |
| 1. Is your choice based on biological pathway? |  |  |
| 1. What do you expect from this medical intervention? |  |  |
| to reduce the jaw volume |  |  |
| to perform dental interventions |  |  |
| to improve aesthetics |  |  |
| to be able to perform surgery, currently impossible |  |  |
| to improve extra-oral involvement |  |  |
| to maintain a putative effect of surgery |  |  |
|  |  |  |
| **DRUG ADMINISTRATION** | **YES** | **NO** |
| 1. Has the molecule been tested in a non-lethal disease on children before? |  |  |
| 1. Have you adapted the dose with body surface or weight? |  |  |
| 1. Have you planned the number of doses to administrate for your expected result? |  |  |
| 1. Have you solicited an external ethics committee review prior to drug administration? |  |  |
| 1. Is your decision for drug therapy independent from any influence other than improving the patient’s health? (family concern, pharmacological conflict of interests…) |  |  |
| 1. Have you identified a clear and measurable outcome to be enhanced with your drug? |  |  |
| 1. Is your outcome measurable? |  |  |
| 1. Is this outcome related to cherubism improvement? (not only a secondary endpoint, e.g. bone sclerosis at the CT-scan evaluation after denosumab) |  |  |
| 1. Have you performed an extensive workup of exams (biological, imaging) dedicated to evaluating bone parameters and potentially drug-modified parameters? |  |  |
| 1. Are the drug’s adverse effects identified and anticipated? |  |  |
| 1. Accordingly, have you anticipated side prescriptions? (e.g. crucial calcium and vitamin D supplements to prevent hypocalcemia with denosumab) |  |  |
|  |  |  |
| **FOLLOW-UP** | **YES** | **NO** |
| 1. Is the first administration performed during a hospitalization stay? |  |  |
| 1. Does the patient and his/her family know how to identify severe side-effects? |  |  |
| 1. Does the follow-up involve radiation exams (iterative CT-scans)? |  |  |
| If yes, could alternatives be considered? |  |  |
| If yes, could the increase of risk be justified? |  |  |
| 1. Are the biomarkers for clinical efficacy of drug therapy clearly identified? |  |  |
| 1. Are the biomarkers for biological efficacy of drug therapy clearly identified? |  |  |
| 1. Is the clinical safety systematically and frequently assessed? |  |  |
| 1. Are the biological parameters for safety management systematically and frequently assessed? |  |  |
| 1. Are reasons for drug interruption anticipated? |  |  |
| 1. Is a clear date for drug cessation planned at baseline? |  |  |
| 1. If the outcome is not reached at the cut-off date, have you planned to stop drug administration? |  |  |
| 1. If no deadline is planned and your outcome is not reached, will you stop drug administration? |  |  |
|  |  |  |
| **DATA SHARING** |  |  |
| 1. Are you interested to share information about your patient with the community of researchers on cherubism? |  |  |

As no further studies have validated this questionnaire, we can only suggest that you answer with a maximum of validated items, rather than a score.
