# Supplementary material for: Pharmacological management of cherubism: A systematic review
Source: Front Endocrinol (Lausanne). 2023 Mar 14;14:1104025. doi: 10.3389/fendo.2023.1104025 (PMC10044089; doi:10.3389/fendo.2023.1104025)
Supplement: Supplementary file 6 [file DataSheet_6.docx]

**References for excluded articles in Appendix 2.**

A.M. Boot, S.M. de Muinck Keizer-Schrama, E.B. Wolvius, Successful calcitonin therapy of two patients with cherubism, Bone. 40 (2007) S29. https://doi.org/10.1016/J.BONE.2007.04.016.

S. Eiden, E. Lausch, S. Meckel, Involution von Cherubismus im MRT unter Therapie mit Imatinib, Rofo. 189 (2017) 675–677. <https://doi.org/10.1055/S-0043-105074>.

G.L. Hart W, Schweitzer DH, Slootweg PJ, [Man with cherubism], Ned Tijdschr Geneeskd. 144 (2000) 34–38.

I. Elimairi, A. Elimairi, A. Sami, A. Salah, The use of calcitonin, denosumab and corticosteroid therapy in the management of bone pathological state (BPS), Int J Oral Maxillofac Surg. 48 (2019) 91. https://doi.org/10.1016/J.IJOM.2019.03.277.

[

Abstracts, Horm Res Paediatr. 91 (2019) 1–682. https://doi.org/10.1159/000501868.

A.Y. Kugushev, A. V. Lopatin, S.A. Yasonov, [Unique Experience of Cherubism Targeted Therapy], Probl Sotsialnoi Gig Zdravookhranenniiai Istor Med. 27 (2019) 608–622. https://doi.org/10.32687/0869-866X-2019-27-SI1-608-622.

A. v. Lopatin, A.Y. Kugushev, S.A. Yasonov, Target therapy of cherubism in a 9 years old child, Pediatric Hematology/Oncology and Immunopathology. 17 (2018) 85–92. https://doi.org/10.24287/1726-1708-2018-17-3-85-92.

M.S. McMahon, Novel treatment of a rare genetic bone disease, Orthopedics. 30 (2007) 91. https://doi.org/10.3928/01477447-20070201-12.

D. MozolováJ. BirčákS. ŠtvrtinaS. Galbavý, (16) Diagnostic and therapeutic problems of cherubism in children and adolescents, (n.d.). https://www.researchgate.net/publication/283138530_Diagnostic_and_therapeutic_problems_of_cherubism_in_children_and_adolescents (accessed July 25, 2022).

W.H. Schreuder, H. van den Berg, J. de Lange, Controversy in the Treatment of Central Giant Cell Granuloma: In Search of Evidence-Based Treatment, Journal of Oral and Maxillofacial Surgery. 69 (2011) e11. https://doi.org/10.1016/J.JOMS.2011.06.231.
